# A review of decision support, risk communication and patient information tools for thrombolytic treatment in acute stroke: lessons for tool developers

**DOI:** 10.1186/1472-6963-13-225

**Published:** 2013-06-18

**Authors:** Darren Flynn, Gary A Ford, Lynne Stobbart, Helen Rodgers, Madeleine J Murtagh, Richard G Thomson

**Affiliations:** 1 Institute of Health and Society, Newcastle University, Newcastle Upon Tyne, UK; 2 Institute for Ageing and Health (Stroke Research Group), Newcastle University, Newcastle Upon Tyne, UK; 3 Department of Health Sciences, University of Leicester, Leicester, UK

**Keywords:** Decision support, Decision aids, Patient information, Shared decision making, Risk communication, Thrombolysis, Acute stroke

## Abstract

**Background:**

Tools to support clinical or patient decision-making in the treatment/management of a health condition are used in a range of clinical settings for numerous preference-sensitive healthcare decisions. Their impact in clinical practice is largely dependent on their quality across a range of domains. We critically analysed currently available tools to support decision making or patient understanding in the treatment of acute ischaemic stroke with intravenous thrombolysis, as an exemplar to provide clinicians/researchers with practical guidance on development, evaluation and implementation of such tools for other preference-sensitive treatment options/decisions in different clinical contexts.

**Methods:**

Tools were identified from bibliographic databases, Internet searches and a survey of UK and North American stroke networks. Two reviewers critically analysed tools to establish: information on benefits/risks of thrombolysis included in tools, and the methods used to convey probabilistic information (verbal descriptors, numerical and graphical); adherence to guidance on presenting outcome probabilities (IPDASi probabilities items) and information content (Picker Institute Checklist); readability (Fog Index); and the extent that tools had comprehensive development processes.

**Results:**

Nine tools of 26 identified included information on a full range of benefits/risks of thrombolysis. Verbal descriptors, frequencies and percentages were used to convey probabilistic information in 20, 19 and 18 tools respectively, whilst nine used graphical methods. Shortcomings in presentation of outcome probabilities (e.g. omitting outcomes without treatment) were identified. Patient information tools had an aggregate median Fog index score of 10. None of the tools had comprehensive development processes.

**Conclusions:**

Tools to support decision making or patient understanding in the treatment of acute stroke with thrombolysis have been sub-optimally developed. Development of tools should utilise mixed methods and strategies to meaningfully involve clinicians, patients and their relatives in an iterative design process; include evidence-based methods to augment interpretability of textual and probabilistic information (e.g. graphical displays showing natural frequencies) on the full range of outcome states associated with available options; and address patients with different levels of health literacy. Implementation of tools will be enhanced when mechanisms are in place to periodically assess the relevance of tools and where necessary, update the mode of delivery, form and information content.

## Background

Clinicians and patients are frequently faced with making a decision about medical or surgical treatment when there are multiple reasonable options (including where appropriate the option of doing nothing) with different balances of short- and long-term benefits, risks and resultant consequences, which are sensitive to individual patient preferences and values [1-3]. Examples of these types of preference-sensitive healthcare decisions/options include treatment of early-stage prostate cancer; screening for genetic conditions; diagnostic tests for suspected gastrointestinal disorders; and medical treatment options for secondary prevention of cardiovascular disease. In contrast to decisions about preference-sensitive care, effective care refers to decisions about treatments that are considered to be a ‘standard’ course of action with largely unequivocal evidence of highly favourable benefit-to-harm ratios, and where consideration of patient/carer preferences and values would add minimal or no value, as there is clearly one superior treatment option [1] For example, repair of fractures and significant lacerations are considered standard [effective] care.

In contrast to decisions about effective care, evidence-based tools such as clinical decision support tools [4], patient decision aids [5], patient information leaflets [6] and risk communication tools [7] are warranted to help clinicians weigh-up the pros and cons of available ‘preference-sensitive’ options by presenting them with evidence-based summaries of the likely magnitude of benefit to risk ratios. Such tools also warranted to (i) promote patient understanding of the available options and probabilistic information on the different balances of benefits, risks and consequences to support choice; and (ii) support clinicians in communicating the latter information to patients, in order to facilitate the process of informed consent, including engagement of patients in shared decision making with clinicians [1]. Tools such as structured patient decision aids, designed specifically to engage patients in shared decision making, impact positively on patients’ knowledge of available options, perception of risk, decisional conflict, clarity about their preferences on available options, patient-practitioner communication and patient involvement in decision making, including reductions in unwarranted variation in rates of preference-sensitive treatment and care [5].

Tools to support clinical and patient decision-making or patient understanding in the treatment/management of a health condition are used in a range of clinical settings for a myriad of preference-sensitive healthcare decisions, although their impact in clinical practice is largely dependent on their quality across a range of domains. Tools should be underpinned by a systematic development process, with reference to evidence-based methods, in order to establish the optimal mode of delivery (electronic or paper-based); form (e.g., graphical methods to convey probabilistic information on outcome states associated with the options); and information content (e.g., comprehensiveness of information on the health condition of interest and available options for treatment, derived from best evidence, including strategies to support health literacy such as readability of textual information and interpretability of probabilistic information by both clinicians and patients) [6,8-13].

The decision to treat or not treat acute ischaemic stroke with intravenous thrombolysis (recombinant tissue plasminogen activator, rt-PA) is a further example of a preference-sensitive healthcare decision, which presents unique challenges for development of tools to support patient understanding / decision making about treatment. Benefit from thrombolysis is time dependent and treatment must be administered within a maximal time window of 4.5 hours from onset of symptoms [14,15]. However, thrombolysis has adverse effects, the most serious being bleeding complications that can cause symptomatic intracranial haemorrhage (SICH) within 24–36 hours following treatment, which usually leads to severe disability or death [16,17], although overall mortality with thrombolysis is not increased and risk of long-term disability significantly reduced [18,19].

Given the extreme time dependent context and the gravity of the potential risks from thrombolysis, clinicians as well as patients (or their relatives/proxy in situations where a patient lacks capacity) are faced with making rapid decisions about treatment that involve deliberation of trade-offs between the increased likelihood of long-term benefit from thrombolysis (reduced risk of significant post-stroke disability) and the more immediate bleeding risks and consequences [20,21]. Health (outcome) states following stroke are sensitive to patient values [22]; however acute stroke is often experienced by patients and relatives as a shocking and traumatic event, which can encumber their understanding of verbal information conveyed by clinicians [23].

Despite evidence of effectiveness, thrombolytic treatment rates for acute stroke are below optimal levels [24]. Other than eligible patients presenting too late to secondary care [25] and limited infrastructure to deliver thrombolysis [26], factors associated with clinical decisions not to offer thrombolytic treatment to patients/relatives include physicians’ uncertainty about administering thrombolysis [27]; physicians' concern about risk of SICH [28]; lack of consensus on relative contraindications [29]; and a lack of data to allow decisions to be supported by differential effectiveness based on individual patient characteristics [30].

A variety of tools may help: to support decision making about thrombolysis; clinicians to convey accurate information on benefits, risks and likely consequences of treating acute ischaemic stroke, with and without thrombolysis to patients/relatives in order to support informed consent; and (where appropriate) engagement of patients/relatives in shared decision making within the emergency (hyperacute) period of stroke.

We critically analysed currently available tools to support decision making or patient understanding in the treatment of acute ischaemic stroke with intravenous thrombolysis, in order to provide clinicians and researchers with practical guidance on development, evaluation and implementation of such tools for other preference-sensitive treatment options/decisions in different clinical contexts.

## Methods

We included currently available paper-based or electronic tools to support decision making, patient understanding and risk communication in acute thrombolytic treatment. Eligible tools had to fulfil the following criteria: focus exclusively on intravenously-administered thrombolysis for treatment of acute ischaemic stroke; include textual or numerical information on risks and benefits of thrombolysis; and written in the English language. Tools were excluded if they focused on arterial thrombolysis or entry in clinical trials.

Included tools were categorised as:

•*Brief decision aids*: designed to guide clinical decision making about thrombolysis, and/or engage patients/relatives in decision making by facilitating understanding of the available options and concomitant risks/benefits.

•*Risk communication tools*: primarily focused on communication of probabilistic information to patients/relatives on risks/benefits of thrombolysis.

•*Patient information tools*: primarily designed for patients/relatives to facilitate understanding of diagnosis, treatment, and management, but not specifically to engage them in decision making [5].

•*Standardised information for clinicians*: primarily designed to support clinicians in explaining the risks/benefits of thrombolysis to patients/relatives.

### Search strategy

Searches (covering the period 1995 to 16^th^ August 2011) were conducted across five bibliographic databases (Medline, EMBASE, PsycINFO, CINAHL and Scopus) using a combination of keywords and MeSH headings. An example of the search strategy can be found in Additional file 1. We also searched the bibliographies of included studies and conducted citation searching using ISI Web of Knowledge.

Key representatives of national stroke networks (UK, North America and Australia) were contacted to disseminate a request to identify tools used nationally or locally to support decision making and risk communication in acute thrombolytic treatment. An Internet search using Google™ was also conducted.

Tools identified were independently screened for eligibility by two reviewers (DF, RGT). Disagreements were resolved through discussion.

### Data extraction and critical analysis

An iterative process, involving pilot testing and discussions between two reviewers (RGT, DF), was used to develop a structured data extraction form.

Data extraction was undertaken independently by both reviewers and disagreements resolved through discussion. The data extraction form captured: title, author(s), country of origin, publication/review date, type of tool (e.g., brief decision aid), format (paper, website, electronic); length (number of A4 pages); and citations in tools to primary or secondary research evidence.

Data were also extracted on: (i) numerical data or a description of acute stroke outcomes included in tools in terms of death; extent of disability (with reference to a widely-used outcome measure in acute stroke care - the modified Rankin Scale [31]); risk of SICH; and impact of SICH following thrombolysis; (ii) methods used to convey probabilistic information on acute stroke outcomes (textual [e.g. verbal descriptors such as ‘higher chance’], numerical [percentages, number needed to treat/harm or frequencies] or graphical risk presentations [e.g., pictograph]), including the use of colour in graphical risk presentations, and red-green colour blind friendliness (absence of concurrent use of red and green); and (iv) adherence to good practice on presentation of outcome probabilities assessed with an 8-item 'probabilities' checklist from the International Patient Decision Aid Standards Instrument (IPADSi) [12]. The IPDASi probabilities checklist has four response options, although during pilot testing difficulties were encountered with grading responses from strongly agree to strongly disagree; consequently response options for the IPDASi probabilities checklist were collapsed into ‘agree’ and ‘disagree’ (coded as 1 and 0 respectively; minimum/maximum score 0 and 8). Comprehensiveness of the development process was assessed with a 6-item checklist based on the Medical Research Council Framework for Design and Evaluation of Complex Interventions [9] and on relevant items from IPDASi [12].

Patient information tools were assessed for: (i) readability using the Fog Index [32] - total number of years in education needed to understand the text: 0.4 × (mean sentence length [number of words divided by the number of sentences] + percentage of hard words) - calculated using an online tool [33]; and (ii) information content (clarity of aims, provision of accurate/unbiased information, facilitating decision making, conflicts of interest, structure/layout and reliability) assessed with a 28-item checklist developed by the Picker Institute [6]. This checklist utilised a 5-point scale ranging from no (score of 1) to yes (score of 5) – for this review these 28 items were coded as ‘agree’ or ‘disagree’ (coded as 1 and 0 respectively; minimum/maximum score 0 and 28).

Based on independent assessment by both reviewers, percentage (raw) agreement between raters was calculated for items in the IPDASi (probabilities), information content and development process checklists. Percentage agreement on the IPDASi (probabilities) items (except for the item on use of natural frequencies [63%]) and development process checklists indicated good/substantial agreement between raters (78% to 100%). Except for items relating to descriptions of the condition (67%) and authors’/developers’ credentials or qualifications (56%), there was a good level of agreement between raters on the information content (Picker Institute) checklist (72% to 100%).

## Results

**Figure 1:**
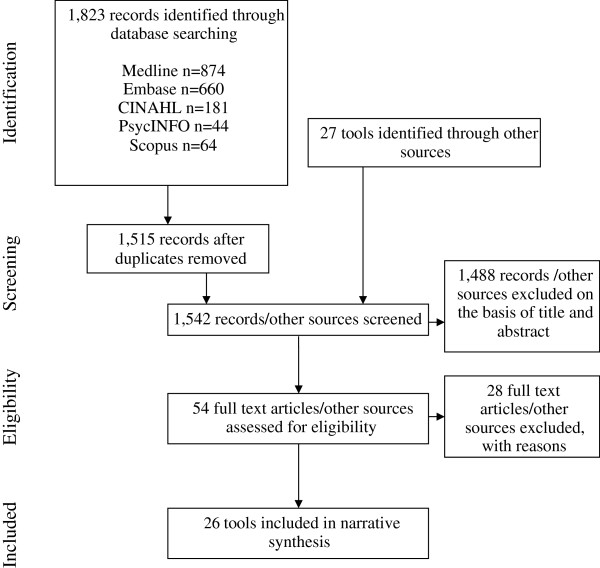
Flowchart summary of the process used to identify tools.

Twenty six tools were identified (Figure 1). Fourteen originated from the UK, nine from the USA, and three from Canada (Table 1). Seventeen were patient information tools [34-50]; five risk communication tools [51-55]; three brief decision aids [56-58]; and one standardised information for clinicians [59]. Patient information and risk communication tools were paper-based (n = 18) or web pages (n = 4). Fifteen of these were equivalent in length to one or two pages of A4; the remainder were equivalent to three or four (n = 4), six or eight (n = 2) or ten pages of A4 (n = 1). All three brief decision aids were electronic tools that used predictive equations to calculate outcomes for individual patients.

**Table 1:** Summary of tools included in the review

**Title**	**Organization/Authors**	**Country**	**Publication date (review date)**	**Type of Tool (research evidence)**	**Format / Length**
HandiStroke: A handheld tool for the emergent evaluation of acute stroke patients	Shapiro et al.	USA	2003 (NS)	Brief decision aid (NINDS)	Electronic/palm-based
The stroke-thrombolytic predictive instrument	Kent et al.	USA	2006 (NS)	Brief decision aid (NINDS 1&2, ATLANTIS A&B, ECASS II)	Electronic/web-based tool
Patient Information Leaflet: Thrombolysis (treatment with a clot dissolving drug - alteplase) for acute stroke	The University of North Staffordshire NHS Trust	UK	2007 (NS)	Patient information tool (NICE)	Paper/1 x A4
After a stroke starts: What you need to know about clot-busting therapy	Genentech	USA	2007 (NS)	Patient information tool (NINDS)	Paper/10 x A4
tPA for stroke: potential benefit, risk and alternatives	American Academy of Emergency Medicine	USA	2007 (NS)	Risk communication tool (NINDS, ECASS I/II, ATLANTIS, MAST-I/E, ASK, Cochrane Review and other literature)	Paper/3 x A4
Clot-busting treatment for acute stroke: patient information	NHS Lothian	UK	2007 (NS)	Risk communication tool (Research evidence NS)	Paper/1 x A4
Clot-busting treatment for acute stroke: patient information	NHS Lothian	UK	2008 (NS)	Risk communication tool Research evidence NS	Paper/1 x A4
Information for Patients about thrombolysis (clot dissolving drugs) for stroke	Royal United Hospital Bath NHS Trust	UK	2008 (Sep 2011)	Patient information tool (Research evidence NS)	Paper/2 x A4
Tissue Plasminogen Activator (tPA) what you should know	American College of Emergency Physicians, American Academy of Neurology, American Heart Association/American Stroke Association	USA	2008 (NS)	Patient information tool (FDA)	Paper/1 x A4
Thrombolytic Treatment of Acute Ischaemic Stroke	Patient UK	UK	2008 (Nov 2011)	Patient information tool (MAST-I/E, ASK, NINDS, ECASS I/II, ATLANTIS, STAT, SITS-MOST, NICE)	Web page/6 x A4
The Outcome Wheel a potential tool for shared decision-making in ischemic stroke thrombolysis	Cunningham	Canada	2008 (NS)	Brief decision aid (NINDS)	Electronic/Excel file
Information to give to patients/relatives before administration of Alteplase	Stroke Northumbria	UK	2009 (NS)	Standardised Information (Research evidence NS)	Paper/< 1 A4
Stroke Thrombolysis (Clot Dissolving Drugs): an information leaflet	Stockport NHS Foundation Trust	UK	2009 (Mar 2010)	Patient information tool (Research evidence NS)	Paper/3 x A4
Information for Patients and families about tPA (Tissue Plasminogen) for Stroke	The Ottawa Hospital	Canada	2009 (NS)	Patient information tool (Research evidence NS)	Paper/1 x A4
Acute Stroke Thrombolysis	University Hospitals Bristol NHS Foundation Trust	UK	2009 (NS)	Patient information tool (Research evidence NS)	Paper/4 x A5
Thrombolysis Treatment after Stroke	Chest, Heart and Stroke Scotland	UK	2009 (NS)	Patient information tool (Research evidence NS)	Paper/16 x A5
Hypothetical representation of 16 patients treated with Activase (t-PA) vs 16 patients treated with placebo is based on NINDS results at 3 months	Genentech	USA	2009 (NS)	Risk communication tool (NINDS 2)	Web page/3 x A4
Alteplase: a treatment for stroke: Information for patients	Knapp et al.	UK	2010 (NS)	Patient information tool (NINDS, ECASS, SITS-MOST)	Paper/4 x A5
Assessment and improvement of figures to visually convey benefit and risk of stroke thrombolysis	Gadhia et al.	USA	2010 (NS)	Risk communication tool (NINDS)	Paper/1 xA4
Patient and carer information leaflet: thrombolysis in stroke	NHS Wales	UK	2010 (expires 2013)	Patient information tool (NICE/SITS-MOST)	Paper/4 x A4
t-PA information sheet	Saint Alphonsus Health System Outreach Program	USA	2010 (NS)	Patient information tool (FDA)	Paper/1 x A4
Patient Information Sheet for Thrombolysis	NHS Fife	UK	2010 (NS)	Patient information tool (Research evidence NS)	Paper/1 x A4
Stroke Thrombolysis Information for Patients and Relatives	Gloucestershire Hospitals NHS Foundation Trust	UK	2011 (NS)	Patient information tool (Research evidence NS)	Web page/2 x A4
Stroke Thrombolysis - Information Sheet	South Tyneside NHS Foundation Trust	UK	2011 (NS)	Patient information tool (Research evidence NS)	Paper/2 x A4
Patient Information Sheet	Massachusetts General Hospital Stroke Service	USA	Unknown (NS)	Patient information tool (FDA)	Paper/1 x A4
Information about tPA	Thunder Bay Regional Health Sciences Centre	Canada	Unknown (NS)	Patient information tool (Research evidence NS)	Web page/1 x A4

NS = not stated.

### Acute stroke outcomes included in tools

Tools that included a description or numerical data on benefits (functional independence), adverse outcomes (dependency or dependency and death combined), death, intracranial haemorrhage (ICH) and the impact of ICH following treatment with thrombolysis are shown in Table 2. Information on independence was included in 25 out of 26 tools. Only 14 out of 26 tools (five out of five risk communication tools, six out of 17 patient information tools, two out of three brief decision aids and the single standardised information for clinicians) included information on dependency (or dependency combined with death). Information on death was included in only 10 out of 26 tools (four out of five risk communication tools, five out of 17 patient information tools and in the standardised information for clinicians). Information on intracranial haemorrhage (ICH) was included in 25 out of 26 tools, with 20 describing the impact of ICH.

**Table 2:** Acute stroke outcomes included in tools

	**Patient information tool (n = 17)**	**Risk communication tool (n = 5)**	**Brief decision aid (n = 3)**	**Standardised information (n = 1)**	**Overall (n = 26)**
Good outcome^*^	17 (100)	5 (100) ^| |^	2 (67)	1 (100)	25 (96)
Poor outcome^**^	2 (12)	5 (100) ^| |^	0 (0)	1 (100)	8 (31)
Poor outcome/death^***^	4 (24)	1 (20) ^| |^	2 (67)	0 (0)	7 (27)
Death	5 (29)	4 (80)	0 (0)	1 (100)	10 (39)
Intra-cranial hemorrhage (ICH)	17 (100)	5 (100) ^| |^	2 (67)	1 (100)	25 (96)
Outcome following ICH	16 (94)	3 (60) ^| |^	0 (0)	1 (100)	20 (77)

Figures are frequencies (percentage frequencies).

* functional independence (no symptoms to slight disability) – approximating to modified Rankin Scale [31] 0 to 1, or 0 to 2.

** dependence (moderate to severe disability) - approximating to modified Rankin Scale 3 to 5.

*** dependence combined with death.

| | one risk communication tool displayed these outcomes using only graphical methods.

Five patient information tools [34,38,41,45,48], three risk communication tools [51-53] and the single standardised information for clinicians [59] included information on both the potential benefits (independence) and the entire range of adverse outcomes following thrombolysis (dependency, death [or dependency combined with death], ICH, and the impact of ICH).

### Methods used to convey probabilistic information

Numerical methods (percentages, frequencies [e.g., “30 out of 100” or “1 in 3 treated patients” have a good outcome] or number needed to treat/harm) used to convey probabilistic information on outcomes are shown in Table 3. Verbal descriptors, frequencies and percentages were used in 20, 19 and 18 tools respectively. A minority (five out of 17) of patient information tools used number needed to treat/harm. Verbal descriptors were a principal feature of patient information tools (16 out of 17), along with frequencies (13 out of 17). Frequencies (five out of five) and percentages (four out of five) were dominant characteristics of risk communication tools. Brief decision aids only included percentages. The standardised information for use by clinicians utilised verbal descriptors and frequencies.

**Table 3:** Methods used to present probabilistic information

	**Textual**	**Numerical**			**Graphical**		
	**Verbal descriptors**	**Percentages**	**Number needed to treat/harm**	**Frequencies**	**Pie chart**	**Bar graph**	**Pictogram/graph**
Patient Information Tool	16 (94)	11 (65)	5 (29)	13 (77)	0 (0)	2 (12)	1 (6)
Risk Communication Tool	3 (60)	4 (80)	0 (0)	5 (100)^*^	1 (20)	0 (0)	4 (80)
Brief Decision Aid	0 (0)	3 (100)	0 (0)	0 (0)	1 (33)	0 (0)	0 (0)
Standardised Information	1 (100)	0 (0)	0 (0)	1 (100)	0 (0)	0 (0)	0 (0)
Overall	20 (77)	18 (69)	5 (19)	19 (73)	2 (8)	2 (8)	5 (19)

Figures are frequencies (percentage frequencies).

* one risk communication tool showed frequencies graphically.

Nine tools used graphical methods. Graphical methods were used in all five risk communication tools [51-55], but infrequently in patient information tools (three out of 17 [34,39,44]) and brief decision aids (one out of three [58]). Colour was a feature in seven graphical risk presentations, but three [34,51,52] could potentially cause perceptual difficulties for people with red-green colour-blindness.

### Readability and information content of patient information tools

Patient information tools were likely to be comprehensible to most patients/relatives with an aggregate median Fog index equivalent to 10 years of education required to understand the text (Table 4). Only six out of 17 patient information tools fulfilled ≥50% of the (total score ≥15 out of 28) Picker Institute criteria [34-36,39,41,46]. Shortcomings were notable in categories relating to conveying accurate information and facilitating decision making, including items on descriptions of the condition, natural course of acute stroke without treatment and acknowledging uncertainty.

**Table 4:** **Information content**[6]**and readability assessment of patient information tools (n = 17)**

**Start with a clear statement of aims?**		**N**	**%**
	Describes its purpose (e.g. to aid decision-making)	9	53
	Describes what it covers (to help the reader judge whether it’s worth carrying on)	8	47
	Describes who it is for (i.e. which patient groups)	15	88
**Provide unbiased and detailed information about options?**			
	Describes the health condition	8	47
	Describes the natural course without treatment	7	41
	Lists the treatment/management/lifestyle options	9	53
	Describes benefits of options	17	100
	Describes risks of options (harms/side-effects/disadvantages)	17	100
	Describes uncertainty around the current evidence (i.e. what is not known)	1	6
	Describes procedures (i.e., treatments, targets, monitoring, behaviour change, etc.)	15	88
**Contain accurate information?**			
	Clearly states the evidence sources used in compiling the information	8	47
	Information quoted is in line with the most up-to-date clinical evidence	8	47
	Where mentioned, prevalence estimates give an accurate impression of how common/rare the condition is	1	6
	Personal opinion and/or advertising are clearly distinguished from evidence-based information	5	29
**Help patients to make appropriate decisions**			
	Acknowledges (explicitly or implicitly) that the patient has decisions to make	8	47
	Helps patients to imagine what it is like to live with the condition and/or treatment effects	4	24
	Asks patients to consider factors (e.g. priorities, motivations, treatment outcomes) affecting possible courses of action	2	12
	Suggests ways and/or provides tools to help patients make decisions	1	6
**Disclose conflicts of interest?**			
	Includes authors’ / developers’ credentials or qualifications	9	53
	Reports source of funding for development and distribution	6	35
**Have a clear structure and layout?**			
	Is consistent in design and layout throughout	17	100
	Includes aids to finding information (e.g. contents, index, site map, or search facility)	5	29
	Important points are emphasised through the use of summaries and/or bullet points	10	59
	Illustrates information with diagrams and/or pictures	7	41
	Where diagrams appear, they are labelled and relate to the subject matter	5	29
	Sections are clearly separated	14	82
**Help the reader judge its reliability**			
	Reports date of publication	10	59
	Includes sources of further information	7	41
**Information content total: median (IQR), min/max**		**13 (6.5), 5/23**	
**Readability (Fog Index): median (IQR), min/max**		**10.0 (2.9), 7.5/15.9**	

### Presentation of outcome probabilities and development process

Risk communication tools attracted the highest median IPDASi (probabilities) total scores (Table 5), with two fulfilling seven or all eight criteria [51,53]. One patient information tool fulfilled seven criteria [34]. Six was the highest total assigned to a brief decision aid [58]. Most tools included probabilities for treatment options (25 out of 26), specified the reference group (25 out of 26) and presented outcomes using frequencies (19 out of 26). Conversely, relatively few included specific time horizons for outcome probabilities (ten out of 26); outcome probabilities for treatment with and without thrombolysis using identical denominators and time horizons (seven out of 26); acknowledgement of uncertainty (three out of 26); multiple methods of viewing probabilities (11 out of 26); or satisfactorily addressed framing bias (eight out of 26).

**Table 5:** IPDASi (probabilities) and development process ratings for tools

	**IPDASi (Probabilities) items **[12]									**Development process items**					
	**1 n (%)**	**2 n (%)**	**3 n (%)**	**4 n (%)**	**5 n (%)**	**6 n (%)**	**7 n (%)**	**8 n (%)**	**Median (IQR) probabilities total**	**1 n (%)**	**2 n (%)**	**3 n (%)**	**4 n (%)**	**5 n (%)**	**6** **n (%)**
RCT	5 (100)	5 (100)	5 (100)	2 (40)	2 (40)	1 (20)	4 (80)	5 (100)	5 (3)	3 (60)	1 (20)	0 (0)	1 (20)	1 (20)	0 (0)
PIT	17 (100)	17 (100)	13 (77)	6 (36)	2 (12)	1 (6)	6 (36)	3 (18)	4 (1.5)	8 (47)	1 (6)	0 (0)	2 (12)	3 (18)	0 (0)
BDA	2 (67)	2 (67)	0 (0)	2 (67)	2 (67)	1 (33)	1 (33)	0 (0)	4 (0)	3 (100)	1 (33)	0 (0)	0 (0)	0 (0)	0 (0)
SI	1 (100)	1 (100)	1 (100)	0 (0)	1 (100)	0 (0)	0 (0)	0 (0)	4 (0)	0 (0)	0 (0)	0 (0)	0 (0)	0 (0)	0 (0)
Overall	25 (96)	25 (96)	19 (73)	10 (39)	7 (27)	3 (12)	11 (42)	8 (31)	4 (2)	14 (54)	3 (12)	0 (0)	3 (12)	4 (15)	0 (0)

RCT = Risk communication tool PIT = Patient information tool BDA = Brief decision aid SI = Standardised information.

*IPDASi (Probabilities) items*: [12].

1 = provides information about outcome probabilities (OPs) associated with the options; 2 = specifies defined group (reference class) of patients for which the OPs apply; 3 = specifies the event rates for OPs (in natural frequencies); 4 = specifies time period over which the OPs apply; 5 = allows the user to compare OPs across options using the same denominator and time period; 6 = provides information about the levels of uncertainty around event/OPs; 7 = provides more than one way of viewing the probabilities; 8 = provides balanced information about event or OPs to limit framing biases.

Development process items:

1 = evidence that developers have considered best available evidence; 2 = intervention based on an established theory/body of evidence; 3 = elicitation study on risk/benefit information required by patients/families or clinicians; 4 = tool subjected to ‘testing’ in patients and families or clinicians; 5 = development of tool guided by steering group; 5 = tool subjected to exploratory trial.

None of the tools fulfilled all six development process criteria (Table 5). Sources of evidence were explicitly cited in 14 out of 26 tools, primarily from randomised controlled trials of thrombolysis (Table 1). Three presented evidence of development informed by established theory or body of evidence [34,52,58]. Usability testing and involvement of steering groups in development was evident for only three [34,41,52] and four [35,36,41,52] tools respectively. None of the tools identified provided evidence that studies had been undertaken to elicit the information needs of users (clinicians, patients or their relatives), or that the tools had been tested in an exploratory trial.

## Discussion

We critically analysed 26 tools that aimed to support decision making or patient understanding in the treatment of acute stroke with thrombolysis, and the majority demonstrated considerable deficiencies. Only nine tools included information on a full range of both benefits and risks of treatment with thrombolysis. The great majority of tools used frequencies to convey probabilistic information on acute stroke outcomes. However, a majority of tools also used verbal descriptors and percentages, which can cause difficulties with understanding and interpretation [60,61]. Furthermore, many tools only presented frequencies for a good outcome, and used verbal descriptors or percentages to convey information on adverse outcomes. Whilst the great majority of tools specified the patient group to which the outcome probabilities applied, they did not compare outcomes with and without thrombolysis, did not use identical denominators and time horizons, and failed to adhere to other good practice guidance on presentation of outcome probabilities. Outcome probabilities were also frequently underpinned by aggregate-level evidence (where stated) on the benefit to harm ratios derived from randomised controlled trials, which may not necessarily reflect outcomes for individual patients within routine practice [62]. Graphical methods were used infrequently to convey probabilistic information, despite evidence that they can enhance risk perception by exploiting rapid (automatic) perceptual capabilities of users [63]. Patient information tools would be understood by the majority of patients/relatives; but not those with low health literacy. It is recommended that health information materials should be written at no higher than 5^th^ to 6^th^ grade reading levels [64,65], in order to maximise universal understanding in the adult population [equating to Fog Index scores of < 7]. Deficiencies in information content of patient information tools were also identified. The great majority of tools lacked development informed by theory or involvement of clinicians, patients and relatives, and were not pilot tested in actual acute stroke settings.

Our findings are not unique to tools designed to support decision-making and patient understanding in the treatment acute stroke with thrombolysis. They reflect those of previous studies in other clinical settings/domains that have identified deficiencies in information content, methods of conveying probabilistic information, and lack of comprehensive development processes [6,66-70].

### Strengths and limitations

The strengths of this review include a comprehensive search strategy and critical appraisal of tools across multiple relevant domains. The authors also have experience of developing and evaluating tools to support decision making about preference-sensitive treatment options, thus adding further context expertise to the review. In terms of weaknesses, we may have missed tools that are unpublished or unavailable via the Internet, although we also surveyed key clinical networks. We may have also missed unpublished information on development processes for tools.

### Implications for research

Our findings have implications that go beyond tools for this clinical setting to other contexts where decision support and risk presentation is being developed. In order to address the shortcomings of tools identified in this review, including analogous tools to support patient understanding and decision making about other preference-sensitive medical or surgical treatment options, tool development should adhere to a structured development process, recommendations of previous research [66-69] and published guidance [6,10-13] in order to: (i) identify the views and perspectives of clinicians and patients/relatives about treatment decision-making about the available options (for example, in-depth interviews or focus groups); (ii) ascertain the complexities of the target clinical setting which may shape decision-making about the available options (for example ethnographic methods); and (iii) understand how tools work in practice (usability testing of alpha prototypes outwith the target clinical setting, and subsequent feasibility testing in the actual clinical setting), and therefore how they may be improved/adapted to the specific context of the clinical setting.

Patient decision aids utilising a structured development process have been described previously; for example to support decision-making about stroke prevention in atrial fibrillation [71]; management of chest pain in the emergency department [72]; and surgical options for breast cancer [73].

Development of tools should utilise mixed methods and appropriate strategies to meaningfully involve clinicians, patients and their relatives in an iterative design process to establish the optimal mode, form and content of tools in the specific context of the clinical setting. Evidence-based methods should also be utilised to augment interpretability of probabilistic information. In particular, presenting a balanced synopsis of the benefits/risks of treatment with and without the available options (and absolute difference in terms of likely benefit or harm) using natural frequencies [61,74,75] with identical denominators and time horizons that relate to a specific reference class [12]. For example, out of 100 patients with acute ischaemic stroke who receive thrombolytic treatment, it is likely that 60, 30 and 10 will be functionally independent, dependent and dead respectively after three months [and there is a risk of SICH in 2 out of every 100 patients treated with thrombolysis]; whereas out of 100 patients not treated with thrombolysis it is likely that 42, 48 and 10 will be functionally independent, dependent and dead respectively after three months – the absolute benefit of treatment with thrombolysis is 18 more patients out of 100 will be independent three months after a stroke).

Graphical displays provide a further mechanism for supporting accurate interpretation of probabilistic information [76], and there is evidence that graphical options such as pictograms/graphs are acceptable to patients irrespective of differences in health literacy [77]. However, the optimal form of graphical display is contentious [76] and we recommend that researchers elicit clinicians' and patients' views and preferences on a range of characteristics of graphical risk presentations during the development process (e.g., format such as bar graphs [clustered or stacked] and pictograms/graphs to convey natural frequencies [with and without specific treatments, including absolute differences], use of colour [being mindful of red-green colour blindness], and order that information on outcomes is presented). Nonetheless, no single method of presenting probabilistic information is likely to be consistent with preferences of all potential users; therefore, multiple methods of presenting probabilistic information is recommended [11-13].

We further advocate that researchers should investigate the potential value of tailoring outcome probabilities to individual patients [13] for enhancing clinical decision making based on individual differential effectiveness in order to aid patients/relatives to understand and consider trade-offs between the benefits, risks and consequences associated with available options and hence make a decision about treatment that is consistent with their preferences and values [13]. However, a prerequisite for tailoring outcomes to individual patients is the availability of robust predictive models that adequately reflect outcomes in routine practice settings. In the absence of suitable predictive models, extensive statistical modelling work is warranted using techniques such as decision analytic modelling [78].

Tools to support patient/carer understanding and decision-making in emergency care contexts are likely to differ from those designed for use in contexts where there is time for protracted discussions, and where patients/carers can access support and resources outside the consultation. In emergency care settings (e.g., treatment decision making about thrombolysis in acute stroke care) usability testing should be undertaken to establish the optimal mode, form and information content of tools for a specific emergency context to enable rapid use by clinicians and patients/carers. Such tools could be used in parallel to other procedures across an emergency care pathway so as to negate/minimise any negative impact on time for decision making and receipt of treatment. In emergency settings, structured decision aids have been shown to be feasible for eliciting patients’ preferences and values about management and treatment options [8].

The cultural sensitivity of tools to support patient understanding and decision making is a further area in need of research. Simply translating tools into multiple languages is unlikely to address the influence of cultural variation on patient preferences and values, or the applicability of tools to support involvement of patients in decision making with clinicians across cultural groups [79].

### Practice implications

The critical appraisal of tools to support decision making or patient understanding about thrombolytic treatment highlights several important implications for implementation of analogous tools in other clinical contexts. Tools with limitations across the multiple domains described in this review are unlikely to be sensitive to the needs of target users and the complexity of the clinical context. Tools with low scores on the IPDASi probabilities items checklist will limit their ability to support patients/carers to make informed values-based decisions. For example, tools that present unbalanced synopses of probabilistic information on outcome states, without appropriate methods of supporting health literacy, may cause clinicians and patients to over- or under-estimate benefits or adverse effects associated with the available options; for example as a result of using percentages to convey single event probabilities [61] (e.g., 60% chance of being independent after a treatment with thrombolysis for acute stroke), relative risk reduction [75], and framing effects [80].

Consequently, tools with significant limitations will diminish their ability to support patient choice, facilitate self-management of illness, improve patient adherence to treatment, and enhance communication processes between clinicians and patients. In the case of decision aids, such limitations will restrict opportunities for patients to clarify their personal preferences and values with regards to the available options - prerequisites for informed values-based decisions [13].

Additional issues that are important for effective implementation of quality tools include training of clinicians to support patient involvement in healthcare and skills development in risk communication, including strategies to support dissemination and social marketing of quality tools [10,81,82]. Relevance and quality of information content of tools may diminish rapidly over time (due to availability of new data on effectiveness of the options for a specific health condition, including changes to clinical practice and information systems that can support delivery of decision support/risk communication), with a concomitant negative impact on uptake rates. Resources and appropriate systems should therefore be in place to periodically assess the relevance of tools and where necessary, update the mode of delivery, form and information content [81].

Undertaking a definitive randomised controlled trial (following a positive exploratory trial) as part of a structured development process has important benefits [9]; however, upon trial completion the relevance and quality of information content for clinical and patient/relative benefit could have diminished. This underpinned our decision to omit an item ‘was the tool subjected to a definitive randomised controlled trial?’ from the development process checklist, and is consistent with the proposal that tools of sufficient quality may be more appropriately tested and further refined within the context of 'real time' service evaluations [10]. There are trade-offs to consider between the pros and cons of these options for implementation of tools following a positive exploratory trial in routine practice settings. A key issue in need of further debate is whether an equivalent of level of evidence required to license or implement psychological, pharmacological or surgical interventions is warranted for implementation of tools of sufficient quality (underpinned by a structured development process and pilot tested in clinical settings, with no apparent risks to safety/adverse effects) to support patient understanding and decision making about these types of interventions.

New tools to support patient understanding and decision making about preference-sensitive healthcare options/decisions are warranted when none currently exist. Tool development is a time- and resource-intensive process, and in cases where numerous tools already exist for a health condition of interest, an optimal strategy would be to adapt currently available tools so they fulfil quality standards [81], informed by a critical evaluation across the multiple domains described in this review, with reference to published guidance [6,10-13].

## Conclusions

Currently available tools to support decision-making or patient understanding in the treatment of acute stroke with thrombolysis have been sub-optimally developed. In particular, the great majority of tools lacked comprehensive development processes; failed to convey information on a full range of range of outcome states; and did not adhere to good practice on presentation of outcome probabilities. This has implications that go beyond tools for this clinical setting to other contexts where decision support and risk presentation is being developed.

The potential impact of tools for supporting decision making and increasing patient understanding of preference-sensitive treatment options will be enhanced when they are (i) underpinned by an auditable structured development process that has meaningfully involved target users throughout the design and testing process; and (ii) utilised evidence-based methods to present a balanced synopsis of probabilistic information on the full range of outcome states, that is understandable and interpretable by patients with different levels of health literacy. Issues such as training/skills development for clinicians and dissemination plans, including strategies to sustain relevance and quality of information content over time, are also key elements in the equation for the effective implementation of tools in clinical settings.

## Endnote

^a^ please contact the corresponding author for copies of these tools.

## Abbreviations

ICH: Intracranial haemorrhage; IPDASi: International patient decision aid standards instrument; rt-PA: Recombinant tissue plasminogen activator; SICH: Symptomatic intracranial haemorrhage.

## Competing interests

LS and MJM have no competing interests to declare. DF, GAF, HR and RGT have been involved in developing a computerised decision aid for thrombolytic treatment in acute stroke care. This may be made available for a cost payable to download the decision aid to cover the costs of technical maintenance and updating of the information content (predictive equations and user interface) in accordance with user feedback and availability of new data on the effectiveness of thrombolysis. GAF’s institution has received research grants from Boehringer Ingelheim (manufacturer of Alteplase), and honoraria from Lundbeck for stroke-related activities. GAF has also received personal remuneration for educational and advisory work from Boehringer Ingelheim and Lundbeck.

## Authors' contributions

RGT, GAF and HR conceived the study. DF conducted the electronic searches. DF and RGT assessed eligibility of tools for inclusion in the review, and conducted data extraction and quality appraisal. All authors provided input to the development of the methods and the drafting process. All authors read and approved the final manuscript.

## Pre-publication history

The pre-publication history for this paper can be accessed here:

http://www.biomedcentral.com/1472-6963/13/225/prepub

## Supplementary Material

Additional file 1 **Electronic search strategy for Medline.** (keywords and MeSH terms). Click here for file
