# Supplementary material for: A review of decision support, risk communication and patient information tools for thrombolytic treatment in acute stroke: lessons for tool developers
Source: BMC Health Serv Res. 2013 Jun 18;13:225. doi: 10.1186/1472-6963-13-225 (PMC3734197; doi:10.1186/1472-6963-13-225)
Supplement: Additional file 1 — Electronic search strategy for Medline. (keywords and MeSH terms). [file 1472-6963-13-225-S1.doc]

**A Review of Decision Support, Risk Communication and Patient Information Tools for Thrombolytic Treatment in Acute Stroke: Lessons for Tool Developers**

**Web Appendix. Electronic Search Strategy for Medline**

|  | exp Stroke/ |
| --- | --- |
|  | thrombol*.mp. |
|  | exp Thrombolytic Therapy/ |
|  | exp Fibrinolytic Agents/ |
|  | exp Tissue Plasminogen Activator/ |
|  | exp Fibrinolysis/ |
|  | 2 ...... or 6 |
|  | 1 and 7 |
|  | exp decision support techniques/ |
|  | exp Decision Making/ |
|  | Decision Support Systems, Clinical/ |
|  | decision aid.mp. |
|  | exp "Sensitivity and Specificity"/ |
|  | exp algorithms/ |
|  | exp Communication/ |
|  | (((((((risk adj3 communicat*) or risk) adj3 present*) or risk) adj2 information) or risk) adj2 perce*).mp. |
|  | (doctor-patient interaction or doctor patient interaction or patient-practitioner communication or patient practitioner communication or patient-caregiver communication or patient care giver communication or patient-physician communication or patient physician communication).mp |
|  | information seeking beh*.mp. |
|  | (informed and (choice or decision or consent)).mp. |
|  | exp Health Education/ |
|  | exp Professional-Family Relations/ |
|  | exp Professional-Patient Relations/ |
|  | exp Patient Participation/ |
|  | (patient and (participation or involvement or information or education or preference or perspective or value)).mp |
| 25. | ((decision-making or decision making) and (shared or sharing or informed or collaborative)).mp. |
|  | 9......or 25 |
|  | 8 and 26 |
|  | limit 27 to (human and English language and yr="1995-Current") |
